# STR analysis of human DNA recovered from bathwater and other water samples for forensic identification

**DOI:** 10.1371/journal.pone.0345878

**Published:** 2026-03-25

**Authors:** Mitsuyo Machida, Kazuhiko Kibayashi

**Affiliations:** Department of Forensic Medicine, School of Medicine, Tokyo Women’s Medical University, Shinjuku-ku, Tokyo, Japan; Universiti Teknologi Malaysia - Main Campus Skudai: Universiti Teknologi Malaysia, MALAYSIA

## Abstract

Human DNA is widely distributed in the environment and can be released into water through skin cells, sweat, and other biological materials. In forensic investigations involving drowning or suspected immersion events, bathwater represents an underexplored but potentially informative source of DNA evidence. This study evaluated the feasibility of recovering and analyzing human DNA from bathwater for short tandem repeat (STR) profiling. Bathwater samples were collected from 11 volunteers after 1, 2, 5, and 10 minutes of immersion, as well as from 12 forensic autopsy cases. DNA was concentrated using a filtration-based method and analyzed for DNA quantity, degradation, and STR profiling success. Both DNA yield and integrity generally increased with immersion duration, and most samples yielded complete STR profiles after 10 minutes. Considerable interindividual variation in DNA shedding was observed, and residual DNA from prior bath users frequently contributed to mixed profiles. Allele drop-ins were common in low-template or mixed samples but decreased as DNA quantity increased. STR profiles were successfully recovered from indoor samples (e.g., bathtubs), whereas no interpretable profiles were obtained in the limited number of outdoor cases examined in this study. These findings demonstrate that bathwater may serve as a useful supplementary source of human DNA in forensic investigations, particularly in indoor drowning cases. Although mixed profiles necessitate cautious interpretation, DNA recovered even after brief immersion may support victim identification or contribute to assessments of potential co-presence in conjunction with other evidence. Overall, this study suggests that bathwater represents a supplementary and context-dependent source of human DNA for forensic analysis in aquatic settings.

## Introduction

Human DNA can be encountered in a wide range of environments, originating from skin cells, sweat, and other biological materials shed during daily activities [1–3]. Individuals are estimated to release 2 × 10^8^–1 × 10^9^ skin cells per day through epidermal desquamation, producing corneocytes that may still contain amplifiable nuclear DNA despite fragmentation [1,4–8]. This DNA has also been identified in household dust and even on insects such as houseflies and cockroaches [9–11]. Moreover, DNA transfer can occur indirectly via clothing or surfaces [12,13]. Collectively, these findings demonstrate that human-derived DNA can persist in common environments and provide material suitable for forensic investigation.

Drowning is a leading cause of unintentional injury and death worldwide, and in Japan, unintentional drowning and submersion represent the second leading cause of death among unintentional injuries, accounting for 21.7% of all unintentional injury-related deaths according to the most recent national statistics (2024) [14]. Such incidents occur not only in swimming pools, rivers, and beaches, but also frequently in household bathtubs [15]. Victims are usually identified at the scene or in the hospital; however, determining whether other individuals were present—particularly in the absence of witnesses—remains a recurring challenge in forensic investigations [16].

Although most bathtub-related deaths are accidental or associated with sudden endogenous conditions, bathtubs have also been implicated in homicides involving forced submersion [17] and in child abuse cases [18–20]. For example, in one documented case, two individuals were immersed together in a bathtub for approximately the same duration; however, STR analysis of the remaining bathwater yielded a complete DNA profile from the deceased individual, whereas the surviving individual’s profile was scarcely detectable. This discrepancy raises important questions about interindividual variation in DNA shedding, degradation, and persistence in aquatic environments.

DNA in aquatic environments has been investigated in several contexts, including environmental DNA (eDNA) research aimed at biodiversity monitoring in open ecosystems such as rivers, lakes, and marine environments [21], as well as forensic studies examining the persistence of human touch DNA and other biological traces on items submerged in water [22]. Most eDNA studies focus on detecting mitochondrial DNA to infer species presence in open aquatic ecosystems, where DNA is frequently diluted and fragmented. Because species detection typically relies on short, high-copy mitochondrial targets, such degradation does not preclude analysis. In contrast, forensic DNA analysis relies primarily on nuclear DNA markers such as STRs for individual identification, for which DNA fragmentation and environmental degradation markedly reduce interpretability. Under these constraints, the properties of the aquatic environment itself become critical for successful forensic DNA analysis. Bathwater therefore represents a fundamentally different aquatic context: a semi-closed, human-dominated environment with a defined water volume, limited exchange, and frequent direct contact with the human body. These features create conditions under which the recovery and interpretation of individual-level human DNA may be more feasible than in open aquatic environments. Accordingly, the forensic interpretation of DNA recovered from bathwater requires empirical evaluation under realistic domestic conditions, particularly under shared or sequential use scenarios, with respect to mixed DNA profiles and the inherent limitations of temporal inference.

Despite its ready availability, bathwater remains largely underexplored in forensic research. Even partial DNA profiles from such samples may provide critical evidence in domestic drowning cases or incidents involving multiple individuals, supporting the establishment of co-presence, corroborating testimonies, or assisting in human identification.

The present study investigates the feasibility of detecting and analyzing human DNA from bathwater using STR profiling. We examine the effects of immersion duration, DNA degradation, and interindividual variation in DNA shedding, using both controlled volunteer experiments and autopsy cases of drowning. Our findings aim to clarify both the potential and the limitations of bathwater and other water samples as a forensic source of human DNA.

## Materials and methods

### Recovery of DNA from bathwater

This study was approved by the Research Ethics Committee of Tokyo Women’s Medical University (Approval No. 2021–0007), and written informed consent was obtained from all participants.

To simulate immersion conditions, 11 healthy Japanese volunteers were recruited between April 25, 2021 and March 21, 2022. Several volunteers belonged to the same household (i.e., family members), and all baths were taken in each volunteer’s household bathtub. In Japan, it is customary to soak in deep bathtubs filled with hot water up to the shoulders for extended periods [23]. Volunteers bathed according to their usual routine, without restrictions on body washing, bathtub cleaning, or water temperature to reflect domestic bathing conditions. To minimize concurrent DNA contribution during immersion, each volunteer bathed individually using newly prepared bathwater.

For each bathing session, 2 L of bathwater was collected in a sterile plastic bottle both before and after immersion for 1, 2, 5, and 10 minutes. The total bathwater volume was calculated from bathtub dimensions (length, width, and water depth), which were measured at the time of bathing. To prevent bacterial growth, 2 mL of 10% benzalkonium chloride solution (FUJIFILM Wako Pure Chemical Corp., Osaka, Japan) was added to each 2-L sample, resulting in a final concentration of 0.01% [24]. Buccal swabs were collected as reference samples using the 4N6 FLOQSwab (4520CS01; Copan Diagnostics, Murrieta, CA, USA).

**Fig 1:**
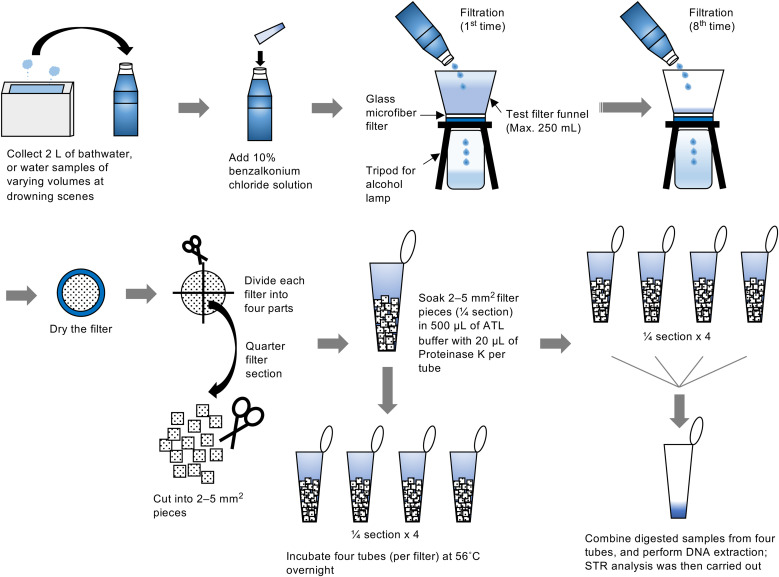
Workflow for recovering DNA from water samples. Bathwater or other water samples were filtered through glass microfiber filters, cut into pieces, digested with Proteinase K, and subjected to DNA extraction, followed by STR analysis. Filtration was repeated until the entire sample volume was processed (up to eight successive filtration cycles).

To minimize contamination, researchers wore laboratory coats, surgical masks, and nitrile gloves, and sterilized all instruments before use. As illustrated in Fig 1, DNA was concentrated by filtration using a sterile test filter funnel (Nalgene 145–2045; Thermo Fisher Scientific, San Jose, CA, USA) equipped with a glass microfiber filter (GF/F 47 mm diameter, pore size, 0.7 µm; 1825–047; GE Healthcare Life Sciences, Chalfont St Giles, Buckinghamshire, UK).

The glass microfiber filter, which has a positively charged surface, has been shown to enhance DNA adsorption [25]. The funnel was mounted on an alcohol lamp tripod (00950035; Niigata Seiki, Niigata, Japan). Because the funnel capacity was 250 mL, each 2-L sample was processed in eight successive filtrations. Filters were air-dried in a biosafety cabinet, wrapped in aluminum foil, and stored at −80°C for up to four months before DNA extraction, as filtration required variable processing times and DNA extraction was performed in batches after sufficient filters had been accumulated.

Scissors used for filter processing were disinfected by immersion in sodium hypochlorite (Kao, Tokyo, Japan) for 20 minutes, followed by UV-C irradiation for 20 minutes. Each filter quarter was cut into 2–5 mm^2^ fragments, which were placed into four separate tubes. Each tube was incubated overnight at 56°C with 500 µL ATL buffer and 20 µL proteinase K (QIAamp DNA Investigator kit; QIAGEN, Venlo, Netherlands) on a ThermoMixer C (Eppendorf, Hamburg, Germany) at 1200 rpm. Following incubation, small filter fragments were removed by centrifugation using DNA IQ Spin Baskets (V1221, Promega, Madison, WI, USA) at 6000 rpm for 1 minute. The combined flow-through was subjected to DNA extraction according to the manufacturer’s protocol (QIAamp DNA Investigator kit: QIAGEN). DNA was eluted in 50 µL of distilled water. DNA quantities were normalized to account for the cumulative bathwater removed (2, 4, 6, or 8 L).

### Evaluation of DNA degradation

DNA quantity and degradation were assessed using the KAPA Human Genomic DNA Quantification and QC Kit (Kapa Biosystems, Wilmington, MA, USA) and the StepOnePlus Real-Time PCR system (Thermo Fisher Scientific). Samples were diluted with UltraPure distilled water (Thermo Fisher Scientific) as required. DNA degradation was evaluated by calculating the ratio of the shorter (41-bp) to the longer (129-bp) amplicons, as determined from Ct values. A ratio close to 1 indicated intact DNA, whereas larger ratios reflected increasing degradation due to the preferential loss of longer fragments. This approach is based on a previous report demonstrating a strong correlation between the 41:129-bp ratio and STR amplification success [26].

### STR analysis

DNA samples were amplified using the AmpFlSTR Identifiler Plus PCR Amplification Kit (Thermo Fisher Scientific) on a GeneAmp 9700 thermal cycler (Thermo Fisher Scientific). Each 12.5-µL reaction contained 5 µL PCR Master Mix, 2.5 µL Primer Mix, and 5 µL template DNA. When available, 0.4 ng of template DNA was used; otherwise, the maximum volume of 5 µL was applied. The cycling protocol comprised an initial denaturation at 95°C for 11 minutes, followed by 29 cycles of denaturation at 94°C for 20 seconds and annealing/extension at 59°C for 3 minutes, with a final extension at 60°C for 10 minutes.

PCR products were mixed with 8.7 µL Hi-Di formamide and 0.3 µL GeneScan LIZ-500 Size Standard, denatured at 95°C for 3 minutes, cooled on ice, and analyzed on a SeqStudio Genetic Analyzer (Thermo Fisher Scientific).

Electropherograms were analyzed using GeneMapper ID-X software ver. 1.4 (Thermo Fisher Scientific). The analytical threshold was set at 175 relative fluorescence units (RFU). Stochastic thresholds were defined as 350 RFU for heterozygous alleles and 750 RFU for homozygous alleles. Preset stutter filters were applied, according to the manufacturer’s recommendations for the AmpFlSTR Identifiler Plus PCR Amplification Kit. Minor peaks located at the locus-specific −1 stutter positions and not exceeding the corresponding kit-provided stutter filter percentages were excluded from interpretation. Allelic peaks not matching the reference profile and exceeding the locus-specific −1 stutter filter percentages were interpreted as originating from individuals other than the bather.

For each sample, STR results were classified on a locus basis using a fixed denominator of 15 autosomal STR loci per sample. Each locus was assigned to one of the following mutually exclusive categories: matching the bather’s reference profile, allelic non-detection, allelic mixture, allele drop-in, or only non-bather locus. Allelic non-detection was defined as the absence of any allele matching the bather’s reference profile above the analytical threshold (175 RFU). Consequently, the sum of all categories equaled 100% for each sample.

### Statistical analysis

Parameters analyzed with respect to immersion duration included the quantity of human DNA recovered, the DNA degradation index, and STR profiling. These outcomes were expressed as the percentage of loci assigned to each interpretation category (matching reference profile, allelic non-detection, allelic mixture, allele drop-in, and only non-bather locus) per sample, based on a fixed denominator of 15 autosomal STR loci. Variables were compared using one-way analysis of variance (ANOVA), and post hoc comparisons were performed using the Steel–Dwass test.

The 95% confidence interval for the median was calculated as:

median ± [1.58 × interquartile range (IQR)/(n)],

where n represents the sample size. The IQR was defined as the difference between the first and third quartiles. All analyses were conducted with JMP software (SAS Institute Inc., Cary, NC, USA). Two independent experimental runs were performed for each volunteer sample, with duplicate measurements per run for DNA quantification and degradation assessment, and consistent STR profiling results were confirmed across runs.

### Forensic autopsy cases

Between February 2008 and April 2023, our department conducted 1,325 forensic autopsies, of which 181 were classified as drowning. In 11 of these cases, DNA profiling was required to support forensic investigations, including assessment of potential co-presence and identification of individuals present at the scene. These included five cases in which victims were transported by emergency services, and six cases involving drowning in non-standard sites (e.g., a neighbor’s pond) or initially unidentified victims.

All 11 deaths were confirmed as drowning, based on autopsy findings including the presence of foam in the trachea, pulmonary edema, and pleural effusion. One case involved a post-burn suicide, where the victim briefly immersed in water after sustaining burns.

Water samples were collected by police personnel as part of routine death investigations, from locations in close proximity to where the body was discovered. In bathtub cases, water was sampled directly from the bathtub. In outdoor cases (e.g., ponds, rivers, swimming pools), water was collected from the surrounding water near the body location at the time of discovery, rather than from the wider perimeter. The timing of water sampling varied depending on case circumstances and was recorded as part of the forensic investigation; estimated immersion duration is summarized in Table 1. All samples were processed using the same filtration-based protocol as in the volunteer study as part of routine death investigations. Subsequently, the DNA analysis results were used in this study after receiving institutional ethical approval. Personal protective equipment (PPE), including N95 masks and nitrile gloves, was used throughout. All procedures related to the autopsy component were approved by the Research Ethics Committee of Tokyo Women’s Medical University (Approval No. 2022–0088), with a waiver of informed consent from the next of kin in accordance with national guidelines. Autopsy-related data used in this study were accessed for research purposes between November 7, 2022 and September 12, 2025, after ethical approval had been granted. All materials were fully pseudonymized, and the investigators did not have access to any directly identifiable information at any stage of data handling, in compliance with national ethical guidelines.

**Table 1:** Cases in which human DNA was extracted from water samples collected at drowning sites.

Case	Age (years)	Sex	Drowning site	Reason for DNA examination	Cause of death	Manner of death	Estimated immersion duration	Water volume (L)	Sample volume (L)
1	80s	F	Pond	Naked	Drowning	Accident	1.5 d	200.0	0.8
2	20s	F	Bathtub	Transported to hospital	Drowning	U.d.	3.0 h	180.0	2.0
3	<10	F	Swimming pool	Transported to hospital	Drowning	Accident	1.0 h	N.e.	2.5
4	80s	F	Bathtub	Transported to hospital	Drowning	U.d.	1.5 h	180.0	2.1
5	80s	M	River	Transported to hospital	Drowning	Suicide	6.0 h	N.e.	5.1
6	50‒60s	M	River	Unknown individual	Drowning	U.d.	1.0 w	N.e.	2.0
7	20s	M	River	Unknown individual	Drowning	U.d.	1.0 w	N.e.	3.4
8	60s	M	River	Unknown individual	Drowning	U.d.	1.0 w	N.e.	2.0
9	60s	M	River	Unknown circumstance	Drowning	U.d.	2.0 d	N.e.	2.0
10	30s	F	Harbor	Unknown individual	Drowning	Suicide	2.0 d	N.e.	3.6
11	40s	M	Harbor	Transported to hospital	Drowning	Accident	5.0 h	N.e.	2.0
12	50s	M	Bathtub	Self-transported	Burn injury	Suicide	< a few min (U.d.)	180.0	2.2

U.d., undetermined; N.e., not examined.

## Results

### Effect of immersion duration on DNA quantity and degradation

The volume of bathwater used by the volunteers ranged from 197.0 to 256.5 L. To estimate the total quantity of human DNA in each bathtub, the DNA quantity recovered from the glass microfiber filters was adjusted based on the ratio between the total bathwater volume and the 2-L sample volume.

**Fig 2:**
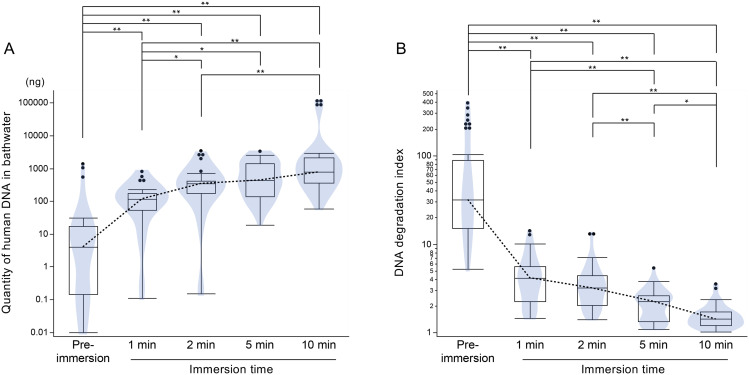
Box–violin plots showing (A) the quantity of human DNA in bathwater and (B) the DNA degradation index at each immersion time. Dotted lines indicate median values. Variables in (A) and (B) are shown on a logarithmic scale. Notches represent the 95% confidence interval for the median. A higher degradation index reflects greater DNA degradation. Statistical significance was assessed by one-way ANOVA followed by the Steel–Dwass test (n = 11, *p < 0.05, **p < 0.01).

A notable increase in human DNA quantity was observed after 1 minute of immersion, followed by a more gradual increase up to 10 minutes (Fig 2A and S1 Table). The DNA degradation index declined with increasing immersion times (Fig 2B and S1 Table). All values were plotted on a logarithmic scale.

### Effect of immersion duration on STR profiling

STR profiling results were classified into five categories:

A. loci at which all detected alleles matched the bather’s reference profile (Matching reference profile),
B. loci at which no allele matching the bather’s reference profile was detected above the analytical threshold (175 RFU) (Allelic non-detection),
C. loci containing a mixture of the bather’s alleles and those from other individuals (Allelic mixtures),
D. loci showing allele drop-ins, defined as alleles not attributable to the bather or any known individual (Allele drop-ins), and
E. loci containing only alleles not attributable to the bather, based on comparison with the bather’s reference profile (Only non-bather loci).

Each locus was assigned to a single category, and percentages were calculated using a fixed denominator of 15 loci per sample.

**Fig 3:**
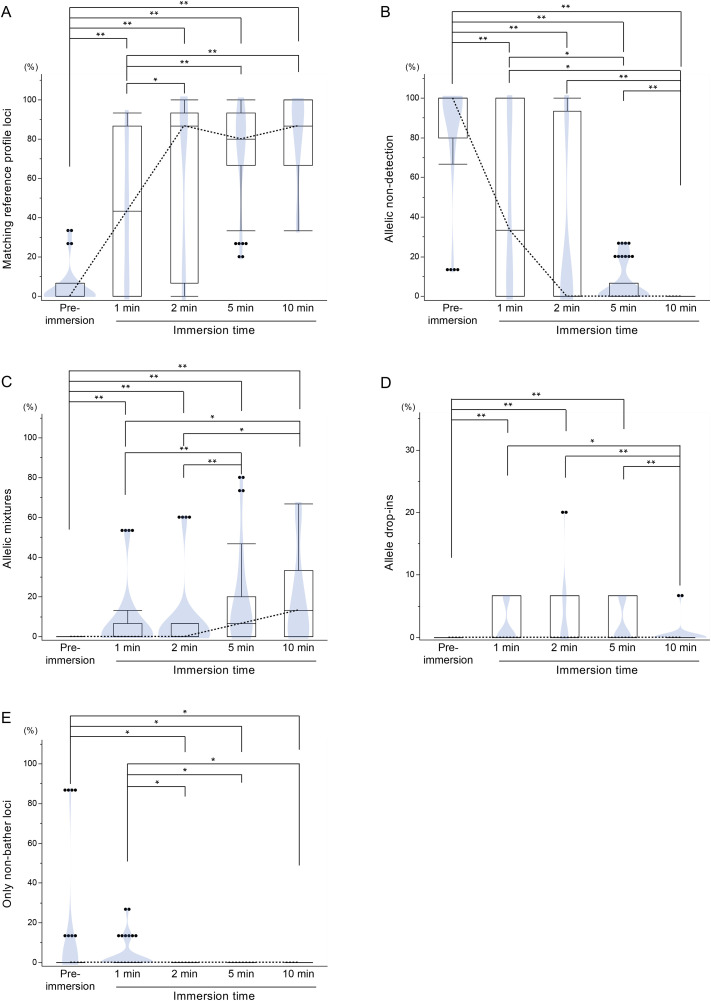
Box–violin plots showing the percentage of loci classified as (A) matching reference profile, (B) allelic non-detection, (C) allelic mixtures, (D) allele drop-ins, and (E) only non-bather loci at each immersion time. Dotted lines indicate median values. Notches represent the 95% confidence interval for the median. Statistical significance was assessed by one-way ANOVA followed by the Steel–Dwass test (n = 11, *p < 0.05, **p < 0.01).

The proportion of loci matching the bather’s reference profile increased significantly after 2 minutes of immersion and reached a plateau by 10 minutes (Fig 3A). Correspondingly, the percentage of loci classified as allelic non-detection declined and was undetectable at 10 minutes (Fig 3B). Allelic mixtures became more frequent after 5 minutes (Fig 3C), while allele drop-ins peaked at 5 minutes and decreased by 10 minutes (Fig 3D). Only non-bather loci were commonly observed in pre-immersion and 1-minute samples (Fig 3E). Mixed profiles were observed in 6, 7, 9, and 7 of the 11 samples collected after 1, 2, 5, and 10 minutes of immersion, respectively. No pull-up artifacts were observed. The raw data underlying Fig 3 are provided in S2 Table. Representative electropherograms (S2–S5 Figures) and their corresponding locus-by-locus interpretations are provided in S5–S8 Tables. A quantitative evaluation of minor allelic peaks interpreted as allelic mixtures or allele drop-ins is provided in S9 Table.

### Interindividual variation in DNA shedding

**Fig 4:**
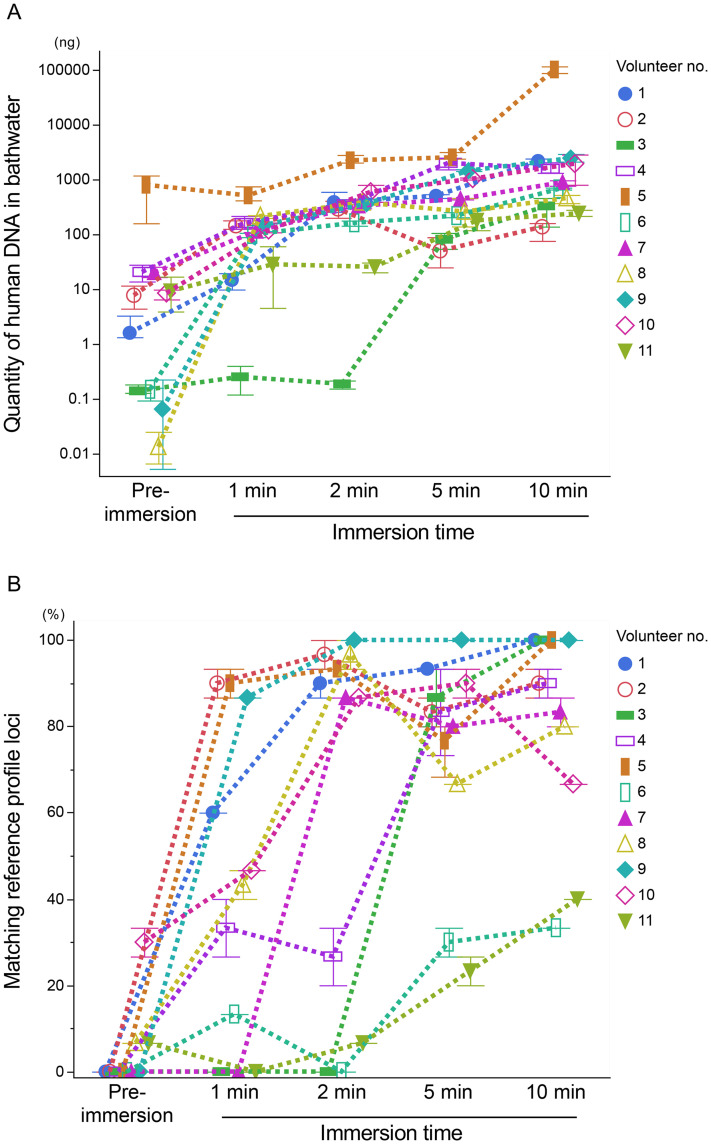
Changes in (A) the quantity of human DNA in bathwater, and (B) the percentage of loci matching the bather’s reference profile before and after immersion for 1, 2, 5, and 10 minutes in each volunteer. Panel (A) is plotted on a logarithmic scale. Symbols represent individual values, error bars indicate the 95% confidence interval for the median, and dotted lines connect the median values across immersion times (n = 11).

To assess interindividual variation in DNA shedding, both the total quantity of human DNA and the accuracy of STR profiling were compared across volunteers. One volunteer (no. 5) consistently exhibited higher DNA quantities across all immersion durations. In contrast, volunteer nos. 3 and 11 showed low DNA quantities at early immersion times (up to 2 minutes), and volunteer nos. 2 and 11 remained low even after 10 minutes (Fig 4A and S3 Table). After 5 minutes of immersion, the proportion of matching reference loci exceeded 80% in 7 of 11 volunteers. Complete STR profiles were obtained from volunteer nos. 1, 3, 5, and 9 after 10 minutes (Fig 4B and S3 Table).

### Autopsy cases

To evaluate the applicability of this method in forensic practice, water samples were collected from 11 drowning cases and one suicide case involving burn injury (case 12). Case information is summarized in Table 1, and the corresponding DNA quantities and STR profiling results are shown in Table 2 (case numbers correspond) and S4 Table.

**Table 2:** Quantities of human DNA, DNA degradation indices, and STR profiling results from water samples collected at drowning sites. Case numbers correspond to those in Table 1.

Case	Human DNA (ng)	DNA degradation index	Matching reference profile loci (%)^a^	Allelic non-detection (%)^b^	Allelic mixtures (%)^c^	Only non-victim loci (%)^d^
1	N.d.	N.d.	0.00 ± 0.00	100.00 ± 0.00	0.00 ± 0.00	0.00 ± 0.00
2	2.70 ± 0.26	1.09 ± 0.10	100.00 ± 0.00	0.00 ± 0.00	0.00 ± 0.00	0.00 ± 0.00
3	0.02 ± 0.00	1.88 ± 0.97	0.00 ± 0.00	100.00 ± 0.00	0.00 ± 0.00	0.00 ± 0.00
4	0.26 ± 0.06	1.32 ± 0.65	100.00 ± 0.00	0.00 ± 0.00	0.00 ± 0.00	0.00 ± 0.00
5	N.d.	N.d.	0.00 ± 0.00	100.00 ± 0.00	0.00 ± 0.00	0.00 ± 0.00
6	< 0.01	< 0.01	0.00 ± 0.00	88.33 ± 3.33	0.00 ± 0.00	11.67 ± 3.33
7	N.d.	N.d.	0.00 ± 0.00	20.00 ± 0.00	40.00 ± 0.00	40.00 ± 0.00
8	N.d.	N.d.	0.00 ± 0.00	90.00 ± 3.85	0.00 ± 0.00	10.00 ± 3.85
9	N.d.	N.d.	0.00 ± 0.00	93.33 ± 0.00	0.00 ± 0.00	6.67 ± 0.00
10	< 0.01	< 0.01	0.00 ± 0.00	100.00 ± 0.00	0.00 ± 0.00	0.00 ± 0.00
11	< 0.01	490.36 ± 9.24	0.00 ± 0.00	100.00 ± 0.00	0.00 ± 0.00	0.00 ± 0.00
12	0.05 ± 0.00	57.54 ± 6.76	23.33 ± 11.55	66.67 ± 12.17	8.33 ± 3.33	1.67 ± 3.33

Values are presented as mean ± S.D. (standard deviation).

N.d., not detected.

^a^Matching the victim’s reference profile loci = loci at which all detected alleles matched the victim’s reference profile;

^b^Allelic non-detection = loci at which no allele matching the victim’s reference profile was detected above the analytical threshold (175 RFU);

^c^Allelic mixtures = loci containing a mixture of the victim’s alleles and those from other individuals;

^d^Only non-victim loci = loci containing only alleles not attributable to the victim.

Extremely high degradation index values exceeding the dynamic range of the assay should be interpreted qualitatively rather than as precise quantitative measures.

The volumes of the collected samples ranged from 0.8 to 5.1 L. Human DNA was detected in six cases: two bathtub drownings (cases 2 and 4), one swimming pool drowning (case 3), and three open-water drownings in a river or harbor (cases 6, 10, and 11). In the open-water cases (cases 6 and 10), the quantity of recovered human DNA was less than 0.01 ng. Notably, bathwater from case 12 yielded DNA in a quantity sufficient for STR analysis. Complete STR profiles were obtained from both individuals in the bathtub drowning cases (cases 2 and 4), whereas only partial (23%) profiles were recovered in case 12. In the limited number of non-bathtub cases examined in this study, including ponds, swimming pools, rivers, and harbors, no STR loci matched reference profiles. Furthermore, in cases 6 and 10, the shorter 41-bp amplicon showed weak signals, whereas in cases 11 and 12, the longer 129-bp amplicon was markedly reduced, indicating severe DNA degradation.

## Discussion

The present study evaluated the efficacy of a filtration-based method for the recovery of human DNA from bathwater. Immersion duration substantially influenced both the quantity and quality of DNA recovered. STR profiling was successful in most samples, particularly after prolonged immersion. These findings support that bathwater can serve as a viable forensic DNA source without direct sampling from the body.

DNA quantity and STR profiling success both increased with immersion time (Fig 2A and Fig 3A). This finding is consistent with a previous study showing that DNA accumulation correlates with the duration of environmental exposure [27]. The release of skin cells and extracellular DNA occurs through physiological processes such as desquamation, sweating, and respiration [1,3,4]. Microscopic examination confirmed abundant corneocytes in bathwater (S1 Figure), which are known to retain amplifiable DNA [6–8]. Bathing further stimulates sweat and sebaceous gland activity [28], leading to the release of cell-free nucleic acids that can support STR profiling [2]. Moreover, the observed decrease in DNA degradation index with prolonged immersion (Fig 2B) suggests greater recovery of intact DNA, likely due to the gradual detachment of inner corneocytes as epidermal swelling progresses [29].

While immersion time was treated as the primary variable in the volunteer experiments, other factors such as bathing behavior, water temperature, mechanical agitation, and pre-existing background DNA were not standardized and may have contributed to interindividual and inter-sample variability. Therefore, the observed relationships between immersion duration and DNA quantity or integrity should be interpreted as associative rather than strictly causal.

Because samples were collected repeatedly from the same individuals across multiple immersion time points, observations were not fully independent. Accordingly, statistical analyses were interpreted descriptively, with emphasis on effect trends rather than strict hypothesis testing.

Alleles originating from individuals other than the current bather were detected both before and after immersion (Fig 3C), indicating the presence of residual DNA. In Japan, it is a common practice for family members to share bathwater, which can lead to the accumulation of biological material such as sebum or corneocytes on bathtub surfaces. These materials may be resuspended during subsequent use [30]. Furthermore, interindividual variation in DNA shedding was evident (Fig 4), consistent with previous reports describing individuals as “good” or “poor” shedders [31–33]. This variability may also explain why residual DNA from high-shedding individuals (e.g., volunteer no. 5) could contaminate samples from low-shedding individuals (e.g., volunteer no. 6), even after water replacement. Such variation may likewise explain situations in which one individual’s DNA profile is readily detectable, whereas another’s is scarcely detectable despite similar immersion conditions.

Allele drop-ins likely represent low-level background DNA or PCR artifacts, occurring more frequently when template DNA quantities were low. These events were observed primarily in samples collected after short immersion (Fig 3D). The reduced frequency of allele drop-ins in samples from longer immersion suggests that increased template DNA availability may mitigate stochastic amplification effects [34]. Consequently, STR profiles obtained after brief immersion require cautious interpretation, whereas prolonged immersion increases the likelihood of mixed profiles due to the accumulation of DNA from multiple individuals.

In forensic practice, STR profiles were successfully recovered from indoor bathwater samples collected during autopsies involving bathtub drownings (Table 2). In contrast, in the limited number of outdoor cases examined in this study, samples obtained from water sources such as ponds, rivers, and swimming pools did not yield interpretable STR profiles. This is likely attributable to environmental factors such as chlorine and disinfection by-products in swimming pools [35], as well as PCR inhibitors including tannins and humic acid in natural waters [36,37]. In addition, ultraviolet radiation can accelerate DNA degradation through strand breakage and cross-linking, while environmental factors such as water flow, microbial activity, and hydrolytic or oxidative processes further impair DNA preservation in aquatic settings [22,38]. These results suggest that while indoor environments provide a controlled setting conducive to forensic DNA analysis, the reliability of this approach is limited in outdoor drowning cases where water quality cannot be controlled. In some autopsy cases, degradation index values exceeded the typical dynamic range of the assay; such values should therefore be interpreted qualitatively as indicators of severe DNA degradation rather than as precise quantitative measures.

The detection of an STR profile in bathwater should not be interpreted as definitive evidence of an individual’s presence at the time of immersion, particularly in shared domestic settings where household dynamics and potential secondary transfer can contribute to mixed DNA profiles [12,13]. The presence of alleles from previous bath users or unidentified individuals complicates interpretation of whether a DNA contributor was physically present during the incident. Although a complete STR profile in bathwater may suggest recent exposure, it does not constitute unequivocal evidence of the individual’s presence, as DNA persistence in bathwater varies with environmental and individual factors and the timing of deposition cannot be reliably determined.

This study demonstrates the forensic potential of bathwater as a non-invasive source of human DNA, particularly in cases of immersion-related death where conventional trace evidence may be scarce. Nevertheless, the frequent occurrence of mixed profiles and the uncertainty regarding the timing of DNA deposition necessitate cautious, case-specific interpretation to ensure reliable forensic conclusions. Bathwater DNA analysis should therefore be regarded as a supplementary line of evidence that may help establish co-presence or support investigative hypotheses, rather than as definitive proof of involvement. The applicability of this approach may also reflect cultural practices, such as the common use of shared bathwater in Japan, and its relevance may be less direct in regions where such habits are uncommon. Although the present study is grounded in the Japanese bathing context, similar scenarios involving shared or sequential use of bathing water may also arise in other settings, such as spas, hotels, assisted living facilities, or other communal bathing environments worldwide. Within the Japanese context, where shared bathing is common, bathwater represents a forensically significant and readily accessible source of DNA. Given the relatively small number of volunteers and autopsy cases analyzed in this study, the present study should be regarded as exploratory, aiming to characterize general trends rather than to establish definitive quantitative thresholds. Despite these limitations, the consistent trends observed provide a foundation for future work. Future studies should expand the sample size, investigate diverse environmental conditions, and assess test reproducibility across broader casework scenarios. In addition, bathing practices that vary across cultures—such as the use of bath foam or other additives—should be examined to better understand their effects on DNA recovery and persistence in bathwater.

## Conclusion

This study demonstrates that human DNA can be recovered from bathwater using a filtration-based method followed by STR profiling. DNA yield and integrity increased with immersion duration, supporting successful STR profiling in bathtub drowning cases. Although mixed profiles and environmental degradation in water sources remain challenges, bathwater DNA analysis represents a useful supplementary tool in forensic investigations involving water immersion. Its practical significance may be greatest in regions where shared bathing is common, such as Japan; however, broader validation under diverse environmental and cultural conditions will be essential to establish international applicability.

## Supporting information

S1 Table Individual data for the quantity of human DNA in bathwater and the DNA degradation index at each immersion time. (PDF)

S2 Table Individual data for the percentage of loci matching the bather’s reference profile (Matching reference profile), loci classified as allelic non-detection (defined as the absence of any allele matching the bather’s reference profile above the analytical threshold of 175 RFU), loci containing a mixture of the bather’s alleles and those from other individuals (Allelic mixtures), loci classified as allele drop-ins (defined as alleles not attributable to the bather or any known individual), and loci containing only alleles not attributable to the bather (Only non-bather loci) at each immersion time. (PDF)

S3 Table Individual data for the quantity of human DNA in bathwater and the percentage of loci matching the bather’s reference profile across immersion times for each volunteer. (PDF)

S4 Table Individual data for the quantity of human DNA, the DNA degradation index, and the STR profiling results from water samples collected at drowning sites. (PDF)

S5 Table Locus-by-locus STR interpretation for a representative bathwater sample collected after 10 minutes of immersion (Volunteer no. 9). (PDF)

S6 Table Locus-by-locus STR interpretation for a representative bathwater sample collected after 5 minutes of immersion (Volunteer no. 5). (PDF)

S7 Table Locus-by-locus STR interpretation for a representative bathwater sample collected after 1 minute of immersion (Volunteer no. 5). (PDF)

S8 Table Locus-by-locus STR interpretation for a representative bathwater sample collected prior to immersion (Volunteer no. 8). (PDF)

S9 Table Quantitative evaluation of minor allelic peaks interpreted as drop-ins or allelic mixtures in S3 and S4 Figs, including peak heights (RFU), stutter ratios, and comparison with locus-specific −1 stutter filter percentages provided in the AmpFlSTR Identifiler Plus PCR Amplification Kit User Guide. **Note:** Two independent runs were performed for each volunteer or water sample, with duplicate measurements per run. Values marked as N.d. (not detected) were excluded from all calculations. Representative electropherograms (S2–S5 Figs) and their corresponding locus-by-locus interpretations are provided in S5–S8 Tables. Quantitative evaluation of minor allelic peaks interpreted as allelic mixtures or allele drop-ins, including peak heights (RFU), stutter ratios, and comparison with locus-specific −1 stutter filter percentages, is provided in S9 Table.
(PDF)

S1 Fig Cells shed in the bathwater stained with Diamond^TM^ nucleic acid dye (DD; left) and hematoxylin and eosin (H&E; right). Upper panels show low-magnification views, and lower panels show high-magnification views. Scale bars: 200 µm (upper), 20 µm (lower). Shed cells were recovered during the final stage of bathwater filtration (at 3–4 mL remaining from each 250 mL fraction) and concentrated to 500 µL using a vacuum dryer.
(PDF)

S2 Fig Representative electropherogram showing a complete STR profile obtained from bathwater collected after 10 minutes of immersion (Volunteer no. 9). All autosomal STR loci matched the reference profile of the bather, indicating a complete STR profile. Amelogenin was excluded from interpretation, as analyses focused on autosomal STR loci.
(PDF)

S3 Fig Representative electropherogram showing allelic mixtures in bathwater collected after 5 minutes of immersion (Volunteer no. 5). Arrows indicate alleles not attributable to the bather, consistent with the definition of allelic mixtures.
(PDF)

S4 Fig Representative electropherogram illustrating allelic mixtures and allele drop-ins in bathwater collected after 1 minute of immersion (Volunteer no. 5). Arrows indicate alleles not attributable to the bather (Allelic mixtures) and alleles not attributable to either the bather or any known individual (Allele drop-ins).
(PDF)

S5 Fig Representative electropherogram obtained from bathwater collected prior to immersion (Volunteer no. 8), showing loci classified as only non-bather loci. Arrows indicate alleles not attributable to the bather. Loci at which only non-bather alleles were detected are indicated below the corresponding loci.
(PDF)
