# Supplementary material for: STR analysis of human DNA recovered from bathwater and other water samples for forensic identification
Source: PLoS One. 2026 Mar 25;21(3):e0345878. doi: 10.1371/journal.pone.0345878 (PMC13016345; doi:10.1371/journal.pone.0345878)
Supplement: S1 Table — (PDF) [file pone.0345878.s001.pdf]

S1 Table. Individual data for the quantity of human DNA in bathwater and the DNA degradation index at each immersion time.

Run	Volunteer no.	Immersion time	Quantity of human DNA in bathwater (ng)	DNA degradation index
Run 1	1	Pre-immersion	1.47	79.77
Run 1	1	Pre-immersion	1.28	91.69
Run 2	1	Pre-immersion	3.79	30.34
Run 2	1	Pre-immersion	1.71	67.43
Run 1	2	Pre-immersion	12.23	22.22
Run 1	2	Pre-immersion	5.99	45.35
Run 2	2	Pre-immersion	9.37	42.02
Run 2	2	Pre-immersion	3.84	102.48
Run 1	3	Pre-immersion	0.15	50.32
Run 1	3	Pre-immersion	0.13	57.84
Run 2	3	Pre-immersion	0.19	36.48
Run 2	3	Pre-immersion	0.13	53.65
Run 1	4	Pre-immersion	29.07	12.06
Run 1	4	Pre-immersion	23.26	15.07
Run 2	4	Pre-immersion	17.49	16.83
Run 2	4	Pre-immersion	12.42	23.71
Run 1	5	Pre-immersion	1204.75	6.30
Run 1	5	Pre-immersion	30.12	5.25
Run 2	5	Pre-immersion	1034.92	8.51
Run 2	5	Pre-immersion	538.16	6.86
Run 1	6	Pre-immersion	0.16	N.d.
Run 1	6	Pre-immersion	0.14	27.54
Run 2	6	Pre-immersion	0.08	21.46
Run 2	6	Pre-immersion	0.17	N.d.
Run 1	7	Pre-immersion	26.29	22.38
Run 1	7	Pre-immersion	22.61	26.02
Run 2	7	Pre-immersion	17.24	33.38
Run 2	7	Pre-immersion	16.20	35.51

Run 1	8	Pre-immersion	0.03	N.d.
Run 1	8	Pre-immersion	0.02	11.47
Run 2	8	Pre-immersion	0.01	N.d.
Run 2	8	Pre-immersion	0.01	9.71
Run 1	9	Pre-immersion	0.01	12.62
Run 1	9	Pre-immersion	0.01	15.02
Run 2	9	Pre-immersion	0.26	9.08
Run 2	9	Pre-immersion	0.12	26.29
Run 1	10	Pre-immersion	7.32	203.55
Run 1	10	Pre-immersion	6.23	391.19
Run 2	10	Pre-immersion	9.73	250.36
Run 2	10	Pre-immersion	7.11	342.96
Run 1	11	Pre-immersion	3.79	223.75
Run 1	11	Pre-immersion	4.17	203.41
Run 2	11	Pre-immersion	17.67	228.87
Run 2	11	Pre-immersion	14.13	286.08
Run 1	1	1 min	20.39	2.92
Run 1	1	1 min	12.85	4.63
Run 2	1	1 min	8.75	2.98
Run 2	1	1 min	16.35	1.59
Run 1	2	1 min	154.22	4.49
Run 1	2	1 min	185.07	3.74
Run 2	2	1 min	122.91	4.71
Run 2	2	1 min	129.06	4.48
Run 1	3	1 min	0.11	4.26
Run 1	3	1 min	0.14	3.28
Run 2	3	1 min	0.41	4.26
Run 2	3	1 min	0.36	4.90
Run 1	4	1 min	138.78	1.58
Run 1	4	1 min	120.74	1.82

Run 2	4	1 min	225.28	1.45
Run 2	4	1 min	177.97	1.84
Run 1	5	1 min	799.39	2.82
Run 1	5	1 min	551.58	4.08
Run 2	5	1 min	454.82	4.93
Run 2	5	1 min	395.69	5.67
Run 1	6	1 min	105.99	3.89
Run 1	6	1 min	85.85	4.81
Run 2	6	1 min	93.77	14.01
Run 2	6	1 min	103.15	12.74
Run 1	7	1 min	114.35	8.31
Run 1	7	1 min	137.23	6.92
Run 2	7	1 min	112.09	9.75
Run 2	7	1 min	107.60	10.16
Run 1	8	1 min	191.08	7.21
Run 1	8	1 min	210.19	6.55
Run 2	8	1 min	213.84	5.40
Run 2	8	1 min	222.40	5.19
Run 1	9	1 min	150.49	1.63
Run 1	9	1 min	142.96	1.72
Run 2	9	1 min	157.94	1.45
Run 2	9	1 min	104.24	2.19
Run 1	10	1 min	173.07	1.64
Run 1	10	1 min	128.07	2.21
Run 2	10	1 min	108.29	3.21
Run 2	10	1 min	107.21	3.25
Run 1	11	1 min	4.31	9.40
Run 1	11	1 min	5.17	6.72
Run 2	11	1 min	62.96	2.42
Run 2	11	1 min	51.00	2.99

Run 1	1	2 min	618.95	2.85
Run 1	1	2 min	488.97	3.61
Run 2	1	2 min	246.90	3.58
Run 2	1	2 min	271.59	3.25
Run 1	2	2 min	224.32	3.46
Run 1	2	2 min	188.43	4.12
Run 2	2	2 min	421.69	2.79
Run 2	2	2 min	350.00	3.36
Run 1	3	2 min	0.22	7.05
Run 1	3	2 min	0.21	7.12
Run 2	3	2 min	0.15	13.39
Run 2	3	2 min	0.16	12.63
Run 1	4	2 min	292.75	1.87
Run 1	4	2 min	322.03	1.70
Run 2	4	2 min	406.07	1.42
Run 2	4	2 min	373.59	1.55
Run 1	5	2 min	2907.86	2.94
Run 1	5	2 min	2297.21	3.72
Run 2	5	2 min	1966.92	3.00
Run 2	5	2 min	2104.61	2.81
Run 1	6	2 min	177.48	4.40
Run 1	6	2 min	170.38	4.58
Run 2	6	2 min	138.81	6.34
Run 2	6	2 min	149.92	5.87
Run 1	7	2 min	338.25	5.01
Run 1	7	2 min	314.58	4.56
Run 2	7	2 min	287.11	5.83
Run 2	7	2 min	356.01	4.42
Run 1	8	2 min	403.11	3.57
Run 1	8	2 min	350.70	4.10

Run 2	8	2 min	349.73	4.11
Run 2	8	2 min	454.65	3.16
Run 1	9	2 min	357.96	1.77
Run 1	9	2 min	340.06	1.86
Run 2	9	2 min	389.60	1.52
Run 2	9	2 min	354.53	1.67
Run 1	10	2 min	360.08	2.46
Run 1	10	2 min	464.50	1.91
Run 2	10	2 min	711.22	2.38
Run 2	10	2 min	810.79	2.08
Run 1	11	2 min	27.56	2.02
Run 1	11	2 min	31.15	1.79
Run 2	11	2 min	19.14	2.91
Run 2	11	2 min	22.97	2.43
Run 1	1	5 min	543.55	1.39
Run 1	1	5 min	494.63	1.53
Run 2	1	5 min	495.48	1.52
Run 2	1	5 min	440.97	1.71
Run 1	2	5 min	18.99	2.64
Run 1	2	5 min	41.78	1.20
Run 2	2	5 min	57.31	2.23
Run 2	2	5 min	97.43	1.31
Run 1	3	5 min	49.31	1.12
Run 1	3	5 min	108.49	1.31
Run 2	3	5 min	66.92	1.12
Run 2	3	5 min	93.69	1.09
Run 1	4	5 min	1850.20	1.42
Run 1	4	5 min	1387.65	1.89
Run 2	4	5 min	2101.15	1.42
Run 2	4	5 min	2563.41	1.16

Run 1	5	5 min	2535.59	3.20
Run 1	5	5 min	3296.27	2.46
Run 2	5	5 min	2329.49	3.54
Run 2	5	5 min	2445.97	3.37
Run 1	6	5 min	200.09	1.28
Run 1	6	5 min	208.10	1.23
Run 2	6	5 min	258.47	1.11
Run 2	6	5 min	224.87	1.28
Run 1	7	5 min	425.52	2.75
Run 1	7	5 min	459.56	2.54
Run 2	7	5 min	431.73	2.33
Run 2	7	5 min	440.37	2.28
Run 1	8	5 min	152.10	5.36
Run 1	8	5 min	132.33	3.83
Run 2	8	5 min	480.57	2.71
Run 2	8	5 min	360.43	3.61
Run 1	9	5 min	1406.64	1.97
Run 1	9	5 min	1448.84	1.91
Run 2	9	5 min	1400.55	2.62
Run 2	9	5 min	1540.61	2.39
Run 1	10	5 min	994.56	2.63
Run 1	10	5 min	1044.28	2.51
Run 2	10	5 min	978.87	2.91
Run 2	10	5 min	1076.75	2.65
Run 1	11	5 min	116.17	2.53
Run 1	11	5 min	120.82	2.43
Run 2	11	5 min	233.71	2.43
Run 2	11	5 min	294.47	1.93
Run 1	1	10 min	2252.68	1.16
Run 1	1	10 min	2410.36	1.08

Run 2	1	10 min	1832.29	1.15
Run 2	1	10 min	2015.52	1.05
Run 1	2	10 min	58.92	1.74
Run 1	2	10 min	123.72	1.29
Run 2	2	10 min	147.04	1.51
Run 2	2	10 min	161.74	1.37
Run 1	3	10 min	101.13	1.43
Run 1	3	10 min	242.71	1.17
Run 2	3	10 min	467.99	1.03
Run 2	3	10 min	407.15	1.19
Run 1	4	10 min	1439.08	1.40
Run 1	4	10 min	1252.00	1.61
Run 2	4	10 min	1643.65	1.26
Run 2	4	10 min	2153.18	1.21
Run 1	5	10 min	114304.05	1.74
Run 1	5	10 min	108588.85	1.83
Run 2	5	10 min	84080.26	2.33
Run 2	5	10 min	89125.07	2.20
Run 1	6	10 min	648.33	1.56
Run 1	6	10 min	674.27	1.50
Run 2	6	10 min	644.58	1.71
Run 2	6	10 min	709.04	1.56
Run 1	7	10 min	958.28	2.29
Run 1	7	10 min	929.53	2.36
Run 2	7	10 min	734.75	3.54
Run 2	7	10 min	822.91	3.16
Run 1	8	10 min	337.81	1.89
Run 1	8	10 min	449.29	1.42
Run 2	8	10 min	454.33	1.64
Run 2	8	10 min	536.10	1.39

Run 1	9	10 min	2066.65	1.80
Run 1	9	10 min	2934.65	1.27
Run 2	9	10 min	2142.52	1.59
Run 2	9	10 min	2678.14	1.49
Run 1	10	10 min	573.58	1.74
Run 1	10	10 min	1433.95	1.07
Run 2	10	10 min	2389.26	1.30
Run 2	10	10 min	2938.79	1.28
Run 1	11	10 min	213.44	1.02
Run 1	11	10 min	279.60	1.11
Run 2	11	10 min	213.44	1.23
Run 2	11	10 min	264.66	1.20

N.d., not detected.
