# Supplementary material for: STR analysis of human DNA recovered from bathwater and other water samples for forensic identification
Source: PLoS One. 2026 Mar 25;21(3):e0345878. doi: 10.1371/journal.pone.0345878 (PMC13016345; doi:10.1371/journal.pone.0345878)
Supplement: S2 Table — (PDF) [file pone.0345878.s002.pdf]

S2 Table. Percentage of loci classified as matching the bather's reference profile, allelic non-detection, allelic mixtures, allele drop-ins, and only non-bather loci at each immersion time.

Run	Volunteer no.	Immersion time	Matching reference profile loci (%)	Allelic non-detection (%)	Allelic mixtures (%)	Allele drop-ins (%)	Only non-bather loci (%)
Run 1	1	Pre-immersion	0.00	100.00	0.00	0.00	0.00
Run 1	1	Pre-immersion	0.00	100.00	0.00	0.00	0.00
Run 1	1	1 min	60.00	20.00	0.00	6.67	13.33
Run 1	1	1 min	60.00	20.00	0.00	6.67	13.33
Run 1	1	2 min	86.67	0.00	6.67	6.67	0.00
Run 1	1	2 min	86.67	0.00	6.67	6.67	0.00
Run 1	1	5 min	93.33	0.00	0.00	6.67	0.00
Run 1	1	5 min	93.33	0.00	0.00	6.67	0.00
Run 1	1	10 min	100.00	0.00	0.00	0.00	0.00
Run 1	1	10 min	100.00	0.00	0.00	0.00	0.00
Run 2	1	Pre-immersion	0.00	100.00	0.00	0.00	0.00
Run 2	1	Pre-immersion	0.00	100.00	0.00	0.00	0.00
Run 2	1	1 min	60.00	20.00	0.00	6.67	13.33
Run 2	1	1 min	60.00	20.00	0.00	6.67	13.33
Run 2	1	2 min	93.33	6.67	0.00	0.00	0.00
Run 2	1	2 min	93.33	6.67	0.00	0.00	0.00
Run 2	1	5 min	93.33	0.00	6.67	0.00	0.00
Run 2	1	5 min	93.33	0.00	6.67	0.00	0.00
Run 2	1	10 min	100.00	0.00	0.00	0.00	0.00
Run 2	1	10 min	100.00	0.00	0.00	0.00	0.00
Run 1	2	Pre-immersion	0.00	100.00	0.00	0.00	0.00
Run 1	2	Pre-immersion	0.00	100.00	0.00	0.00	0.00
Run 1	2	1 min	86.67	13.33	0.00	0.00	0.00
Run 1	2	1 min	86.67	13.33	0.00	0.00	0.00
Run 1	2	2 min	93.33	0.00	0.00	6.67	0.00
Run 1	2	2 min	93.33	0.00	0.00	6.67	0.00
Run 1	2	5 min	86.67	0.00	13.33	0.00	0.00

Run 1	2	5 min	86.67	0.00	13.33	0.00	0.00
Run 1	2	10 min	93.33	0.00	0.00	6.67	0.00
Run 1	2	10 min	93.33	0.00	0.00	6.67	0.00
Run 2	2	Pre-immersion	0.00	100.00	0.00	0.00	0.00
Run 2	2	Pre-immersion	0.00	100.00	0.00	0.00	0.00
Run 2	2	1 min	93.33	6.67	0.00	0.00	0.00
Run 2	2	1 min	93.33	6.67	0.00	0.00	0.00
Run 2	2	2 min	100.00	0.00	0.00	0.00	0.00
Run 2	2	2 min	100.00	0.00	0.00	0.00	0.00
Run 2	2	5 min	80.00	6.67	6.67	6.67	0.00
Run 2	2	5 min	80.00	6.67	6.67	6.67	0.00
Run 2	2	10 min	86.67	0.00	13.33	0.00	0.00
Run 2	2	10 min	86.67	0.00	13.33	0.00	0.00
Run 1	3	Pre-immersion	0.00	100.00	0.00	0.00	0.00
Run 1	3	Pre-immersion	0.00	100.00	0.00	0.00	0.00
Run 1	3	1 min	0.00	100.00	0.00	0.00	0.00
Run 1	3	1 min	0.00	100.00	0.00	0.00	0.00
Run 1	3	2 min	0.00	100.00	0.00	0.00	0.00
Run 1	3	2 min	0.00	100.00	0.00	0.00	0.00
Run 1	3	5 min	80.00	20.00	0.00	0.00	0.00
Run 1	3	5 min	80.00	20.00	0.00	0.00	0.00
Run 1	3	10 min	100.00	0.00	0.00	0.00	0.00
Run 1	3	10 min	100.00	0.00	0.00	0.00	0.00
Run 2	3	Pre-immersion	0.00	100.00	0.00	0.00	0.00
Run 2	3	Pre-immersion	0.00	100.00	0.00	0.00	0.00
Run 2	3	1 min	0.00	100.00	0.00	0.00	0.00
Run 2	3	1 min	0.00	100.00	0.00	0.00	0.00
Run 2	3	2 min	0.00	100.00	0.00	0.00	0.00
Run 2	3	2 min	0.00	100.00	0.00	0.00	0.00
Run 2	3	5 min	93.33	6.67	0.00	0.00	0.00

Run 2	3	5 min	93.33	6.67	0.00	0.00	0.00
Run 2	3	10 min	100.00	0.00	0.00	0.00	0.00
Run 2	3	10 min	100.00	0.00	0.00	0.00	0.00
Run 1	4	Pre-immersion	0.00	100.00	0.00	0.00	0.00
Run 1	4	Pre-immersion	0.00	100.00	0.00	0.00	0.00
Run 1	4	1 min	40.00	20.00	6.67	6.67	26.67
Run 1	4	1 min	40.00	20.00	6.67	6.67	26.67
Run 1	4	2 min	20.00	0.00	60.00	20.00	0.00
Run 1	4	2 min	20.00	0.00	60.00	20.00	0.00
Run 1	4	5 min	93.33	0.00	6.67	0.00	0.00
Run 1	4	5 min	93.33	0.00	6.67	0.00	0.00
Run 1	4	10 min	93.33	0.00	6.67	0.00	0.00
Run 1	4	10 min	93.33	0.00	6.67	0.00	0.00
Run 2	4	Pre-immersion	0.00	100.00	0.00	0.00	0.00
Run 2	4	Pre-immersion	0.00	100.00	0.00	0.00	0.00
Run 2	4	1 min	26.67	46.67	13.33	0.00	13.33
Run 2	4	1 min	26.67	46.67	13.33	0.00	13.33
Run 2	4	2 min	33.33	0.00	60.00	6.67	0.00
Run 2	4	2 min	33.33	0.00	60.00	6.67	0.00
Run 2	4	5 min	73.33	0.00	20.00	6.67	0.00
Run 2	4	5 min	73.33	0.00	20.00	6.67	0.00
Run 2	4	10 min	86.67	0.00	13.33	0.00	0.00
Run 2	4	10 min	86.67	0.00	13.33	0.00	0.00
Run 1	5	Pre-immersion	0.00	13.33	0.00	0.00	86.67
Run 1	5	Pre-immersion	0.00	13.33	0.00	0.00	86.67
Run 1	5	1 min	86.67	0.00	6.67	6.67	0.00
Run 1	5	1 min	86.67	0.00	6.67	6.67	0.00
Run 1	5	2 min	93.33	0.00	0.00	6.67	0.00
Run 1	5	2 min	93.33	0.00	0.00	6.67	0.00
Run 1	5	5 min	66.67	0.00	33.33	0.00	0.00

Run 1	5	5 min	80.00	0.00	20.00	0.00	0.00
Run 1	5	10 min	100.00	0.00	0.00	0.00	0.00
Run 1	5	10 min	100.00	0.00	0.00	0.00	0.00
Run 2	5	Pre-immersion	0.00	13.33	0.00	0.00	86.67
Run 2	5	Pre-immersion	0.00	13.33	0.00	0.00	86.67
Run 2	5	1 min	93.33	0.00	0.00	6.67	0.00
Run 2	5	1 min	93.33	0.00	0.00	6.67	0.00
Run 2	5	2 min	93.33	0.00	0.00	6.67	0.00
Run 2	5	2 min	93.33	0.00	0.00	6.67	0.00
Run 2	5	5 min	73.33	0.00	26.67	0.00	0.00
Run 2	5	5 min	80.00	0.00	20.00	0.00	0.00
Run 2	5	10 min	100.00	0.00	0.00	0.00	0.00
Run 2	5	10 min	100.00	0.00	0.00	0.00	0.00
Run 1	6	Pre-immersion	0.00	100.00	0.00	0.00	0.00
Run 1	6	Pre-immersion	0.00	100.00	0.00	0.00	0.00
Run 1	6	1 min	13.33	86.67	0.00	0.00	0.00
Run 1	6	1 min	13.33	86.67	0.00	0.00	0.00
Run 1	6	2 min	0.00	100.00	0.00	0.00	0.00
Run 1	6	2 min	0.00	100.00	0.00	0.00	0.00
Run 1	6	5 min	26.67	20.00	46.67	6.67	0.00
Run 1	6	5 min	26.67	20.00	46.67	6.67	0.00
Run 1	6	10 min	33.33	0.00	66.67	0.00	0.00
Run 1	6	10 min	33.33	0.00	66.67	0.00	0.00
Run 2	6	Pre-immersion	0.00	100.00	0.00	0.00	0.00
Run 2	6	Pre-immersion	0.00	100.00	0.00	0.00	0.00
Run 2	6	1 min	13.33	86.67	0.00	0.00	0.00
Run 2	6	1 min	13.33	86.67	0.00	0.00	0.00
Run 2	6	2 min	0.00	100.00	0.00	0.00	0.00
Run 2	6	2 min	0.00	100.00	0.00	0.00	0.00
Run 2	6	5 min	33.33	20.00	40.00	6.67	0.00

Run 2	6	5 min	33.33	20.00	40.00	6.67	0.00
Run 2	6	10 min	33.33	0.00	66.67	0.00	0.00
Run 2	6	10 min	33.33	0.00	66.67	0.00	0.00
Run 1	7	Pre-immersion	0.00	100.00	0.00	0.00	0.00
Run 1	7	Pre-immersion	0.00	100.00	0.00	0.00	0.00
Run 1	7	1 min	0.00	100.00	0.00	0.00	0.00
Run 1	7	1 min	0.00	100.00	0.00	0.00	0.00
Run 1	7	2 min	86.67	6.67	6.67	0.00	0.00
Run 1	7	2 min	86.67	6.67	6.67	0.00	0.00
Run 1	7	5 min	80.00	0.00	20.00	0.00	0.00
Run 1	7	5 min	80.00	0.00	20.00	0.00	0.00
Run 1	7	10 min	80.00	0.00	20.00	0.00	0.00
Run 1	7	10 min	80.00	0.00	20.00	0.00	0.00
Run 2	7	Pre-immersion	0.00	100.00	0.00	0.00	0.00
Run 2	7	Pre-immersion	0.00	100.00	0.00	0.00	0.00
Run 2	7	1 min	0.00	100.00	0.00	0.00	0.00
Run 2	7	1 min	0.00	100.00	0.00	0.00	0.00
Run 2	7	2 min	86.67	6.67	6.67	0.00	0.00
Run 2	7	2 min	86.67	6.67	6.67	0.00	0.00
Run 2	7	5 min	80.00	0.00	20.00	0.00	0.00
Run 2	7	5 min	80.00	0.00	20.00	0.00	0.00
Run 2	7	10 min	86.67	0.00	13.33	0.00	0.00
Run 2	7	10 min	86.67	0.00	13.33	0.00	0.00
Run 1	8	Pre-immersion	6.67	80.00	0.00	0.00	13.33
Run 1	8	Pre-immersion	6.67	80.00	0.00	0.00	13.33
Run 1	8	1 min	46.67	53.33	0.00	0.00	0.00
Run 1	8	1 min	46.67	53.33	0.00	0.00	0.00
Run 1	8	2 min	93.33	0.00	6.67	0.00	0.00
Run 1	8	2 min	93.33	0.00	6.67	0.00	0.00
Run 1	8	5 min	66.67	26.67	6.67	0.00	0.00

Run 1	8	5 min	66.67	26.67	6.67	0.00	0.00
Run 1	8	10 min	80.00	0.00	20.00	0.00	0.00
Run 1	8	10 min	80.00	0.00	20.00	0.00	0.00
Run 2	8	Pre-immersion	6.67	80.00	0.00	0.00	13.33
Run 2	8	Pre-immersion	6.67	80.00	0.00	0.00	13.33
Run 2	8	1 min	40.00	53.33	0.00	6.67	0.00
Run 2	8	1 min	40.00	53.33	0.00	6.67	0.00
Run 2	8	2 min	100.00	0.00	0.00	0.00	0.00
Run 2	8	2 min	100.00	0.00	0.00	0.00	0.00
Run 2	8	5 min	66.67	26.67	6.67	0.00	0.00
Run 2	8	5 min	66.67	26.67	6.67	0.00	0.00
Run 2	8	10 min	80.00	0.00	20.00	0.00	0.00
Run 2	8	10 min	80.00	0.00	20.00	0.00	0.00
Run 1	9	Pre-immersion	0.00	100.00	0.00	0.00	0.00
Run 1	9	Pre-immersion	0.00	100.00	0.00	0.00	0.00
Run 1	9	1 min	86.67	0.00	13.33	0.00	0.00
Run 1	9	1 min	86.67	0.00	13.33	0.00	0.00
Run 1	9	2 min	100.00	0.00	0.00	0.00	0.00
Run 1	9	2 min	100.00	0.00	0.00	0.00	0.00
Run 1	9	5 min	100.00	0.00	0.00	0.00	0.00
Run 1	9	5 min	100.00	0.00	0.00	0.00	0.00
Run 1	9	10 min	100.00	0.00	0.00	0.00	0.00
Run 1	9	10 min	100.00	0.00	0.00	0.00	0.00
Run 2	9	Pre-immersion	0.00	100.00	0.00	0.00	0.00
Run 2	9	Pre-immersion	0.00	100.00	0.00	0.00	0.00
Run 2	9	1 min	86.67	0.00	13.33	0.00	0.00
Run 2	9	1 min	86.67	0.00	13.33	0.00	0.00
Run 2	9	2 min	100.00	0.00	0.00	0.00	0.00
Run 2	9	2 min	100.00	0.00	0.00	0.00	0.00
Run 2	9	5 min	100.00	0.00	0.00	0.00	0.00

Run 2	9	5 min	100.00	0.00	0.00	0.00	0.00
Run 2	9	10 min	100.00	0.00	0.00	0.00	0.00
Run 2	9	10 min	100.00	0.00	0.00	0.00	0.00
Run 1	10	Pre-immersion	33.33	66.67	0.00	0.00	0.00
Run 1	10	Pre-immersion	33.33	66.67	0.00	0.00	0.00
Run 1	10	1 min	46.67	0.00	53.33	0.00	0.00
Run 1	10	1 min	46.67	0.00	53.33	0.00	0.00
Run 1	10	2 min	86.67	0.00	6.67	6.67	0.00
Run 1	10	2 min	86.67	0.00	6.67	6.67	0.00
Run 1	10	5 min	86.67	0.00	6.67	6.67	0.00
Run 1	10	5 min	86.67	0.00	6.67	6.67	0.00
Run 1	10	10 min	66.67	0.00	33.33	0.00	0.00
Run 1	10	10 min	66.67	0.00	33.33	0.00	0.00
Run 2	10	Pre-immersion	26.67	73.33	0.00	0.00	0.00
Run 2	10	Pre-immersion	26.67	73.33	0.00	0.00	0.00
Run 2	10	1 min	46.67	0.00	53.33	0.00	0.00
Run 2	10	1 min	46.67	0.00	53.33	0.00	0.00
Run 2	10	2 min	86.67	0.00	6.67	6.67	0.00
Run 2	10	2 min	86.67	0.00	6.67	6.67	0.00
Run 2	10	5 min	93.33	0.00	0.00	6.67	0.00
Run 2	10	5 min	93.33	0.00	0.00	6.67	0.00
Run 2	10	10 min	66.67	0.00	33.33	0.00	0.00
Run 2	10	10 min	66.67	0.00	33.33	0.00	0.00
Run 1	11	Pre-immersion	6.67	93.33	0.00	0.00	0.00
Run 1	11	Pre-immersion	6.67	93.33	0.00	0.00	0.00
Run 1	11	1 min	0.00	100.00	0.00	0.00	0.00
Run 1	11	1 min	0.00	100.00	0.00	0.00	0.00
Run 1	11	2 min	6.67	93.33	0.00	0.00	0.00
Run 1	11	2 min	6.67	93.33	0.00	0.00	0.00
Run 1	11	5 min	26.67	0.00	73.33	0.00	0.00

Run 1	11	5 min	26.67	0.00	73.33	0.00	0.00
Run 1	11	10 min	40.00	0.00	60.00	0.00	0.00
Run 1	11	10 min	40.00	0.00	60.00	0.00	0.00
Run 2	11	Pre-immersion	6.67	93.33	0.00	0.00	0.00
Run 2	11	Pre-immersion	6.67	93.33	0.00	0.00	0.00
Run 2	11	1 min	0.00	100.00	0.00	0.00	0.00
Run 2	11	1 min	0.00	100.00	0.00	0.00	0.00
Run 2	11	2 min	6.67	93.33	0.00	0.00	0.00
Run 2	11	2 min	6.67	93.33	0.00	0.00	0.00
Run 2	11	5 min	20.00	0.00	80.00	0.00	0.00
Run 2	11	5 min	20.00	0.00	80.00	0.00	0.00
Run 2	11	10 min	40.00	0.00	60.00	0.00	0.00
Run 2	11	10 min	40.00	0.00	60.00	0.00	0.00

Percentages were calculated on a locus basis using a fixed denominator of 15 autosomal STR loci per sample; thus, the sum of all categories equaled 100% for each sample.

Allelic non-detection (%) refers to loci at which no allele matching the bather's reference profile was detected above the analytical threshold (175 RFU).

For pre-immersion samples, 100% non-detection reflects the absence of detectable alleles and does not represent allelic dropout from an expected STR profile.

Allele drop-ins were defined as alleles not attributable to the bather or any known individual.

Only non-bather loci refer to loci containing exclusively alleles not matching the bather's reference profile.
