# Supplementary material for: STR analysis of human DNA recovered from bathwater and other water samples for forensic identification
Source: PLoS One. 2026 Mar 25;21(3):e0345878. doi: 10.1371/journal.pone.0345878 (PMC13016345; doi:10.1371/journal.pone.0345878)
Supplement: S4 Table — (PDF) [file pone.0345878.s004.pdf]

S4 Table. Individual data of the quantities of human DNA, the DNA degradation index, and the STR profiling results from water samples collected at drowning sites.

Run	Case	Human DNA (ng)	DNA degradation index	Matching reference profile loci (%)	Allelic non-detection (%)	Allele mixtures (%)	Only non-victim loci (%)
Run 1	1	N.d.	N.d.	0.00	100.00	0.00	0.00
Run 1	1	N.d.	N.d.	0.00	100.00	0.00	0.00
Run 2	1	N.d.	N.d.	0.00	100.00	0.00	0.00
Run 2	1	N.d.	N.d.	0.00	100.00	0.00	0.00
Run 1	2	2.46	0.95	100.00	0.00	0.00	0.00
Run 1	2	2.78	1.20	100.00	0.00	0.00	0.00
Run 2	2	3.03	1.12	100.00	0.00	0.00	0.00
Run 2	2	2.53	1.10	100.00	0.00	0.00	0.00
Run 1	3	0.02	0.81	0.00	100.00	0.00	0.00
Run 1	3	N.d.	N.d.	0.00	100.00	0.00	0.00
Run 2	3	0.02	2.69	0.00	100.00	0.00	0.00
Run 2	3	0.02	2.14	0.00	100.00	0.00	0.00
Run 1	4	0.20	2.18	100.00	0.00	0.00	0.00
Run 1	4	0.27	0.69	100.00	0.00	0.00	0.00
Run 2	4	0.34	0.99	100.00	0.00	0.00	0.00
Run 2	4	0.23	1.43	100.00	0.00	0.00	0.00
Run 1	5	N.d.	N.d.	0.00	100.00	0.00	0.00
Run 1	5	N.d.	N.d.	0.00	100.00	0.00	0.00
Run 2	5	N.d.	N.d.	0.00	100.00	0.00	0.00
Run 2	5	N.d.	N.d.	0.00	100.00	0.00	0.00
Run 1	6	1.1E-05	0.00015	0.00	86.67	0.00	13.33
Run 1	6	1.0E-05	0.00031	0.00	93.33	0.00	6.67
Run 2	6	1.0E-05	0.00050	0.00	86.67	0.00	13.33
Run 2	6	1.1E-05	0.00026	0.00	86.67	0.00	13.33
Run 1	7	N.d.	N.d.	0.00	20.00	40.00	40.00
Run 1	7	N.d.	N.d.	0.00	20.00	40.00	40.00
Run 2	7	N.d.	N.d.	0.00	20.00	40.00	40.00

Run 2	7	N.d.	N.d.	0.00	20.00	40.00	40.00
Run 1	8	N.d.	N.d.	0.00	86.67	0.00	13.33
Run 1	8	N.d.	N.d.	0.00	86.67	0.00	13.33
Run 2	8	N.d.	N.d.	0.00	93.33	0.00	6.67
Run 2	8	N.d.	N.d.	0.00	93.33	0.00	6.67
Run 1	9	N.d.	N.d.	0.00	93.33	0.00	6.67
Run 1	9	N.d.	N.d.	0.00	93.33	0.00	6.67
Run 2	9	N.d.	N.d.	0.00	93.33	0.00	6.67
Run 2	9	N.d.	N.d.	0.00	93.33	0.00	6.67
Run 1	10	2.2E-05	0.00044	0.00	100	0.00	0.00
Run 1	10	1.8E-05	0.00042	0.00	100	0.00	0.00
Run 2	10	1.9E-05	0.00044	0.00	100	0.00	0.00
Run 2	10	2.2E-05	0.00057	0.00	100	0.00	0.00
Run 1	11	N.d.	N.d.	0.00	100	0.00	0.00
Run 1	11	1.3E-05	482.62	0.00	100	0.00	0.00
Run 2	11	1.5E-05	500.59	0.00	100	0.00	0.00
Run 2	11	1.2E-05	487.86	0.00	100	0.00	0.00
Run 1	12	0.04	64.74	13.33	80.00	6.67	0.00
Run 1	12	0.05	53.26	13.33	73.33	6.67	6.67
Run 2	12	0.05	50.47	33.33	53.33	13.33	0.00
Run 2	12	0.04	61.68	33.33	60.00	6.67	0.00

N.d., not detected.
