# Supplementary material for: STR analysis of human DNA recovered from bathwater and other water samples for forensic identification
Source: PLoS One. 2026 Mar 25;21(3):e0345878. doi: 10.1371/journal.pone.0345878 (PMC13016345; doi:10.1371/journal.pone.0345878)
Supplement: S5 Table — (PDF) [file pone.0345878.s005.pdf]

S5 Table. Locus-by-locus STR interpretation for a representative bathwater sample collected after 10 minutes of immersion (Volunteer no. 9).

Locus	Reference genotype	Observed in bathwater	Interpretation
D8S1179	13, 15	13, 15	Matching reference profile locus
D21S11	31.2, 32.2	31.2, 32.2	Matching reference profile locus
D7S820	12	12	Matching reference profile locus
CSF1PO	10, 11	10, 11	Matching reference profile locus
D3S1358	14, 15	14, 15	Matching reference profile locus
TH01	9	9	Matching reference profile locus
D13S317	8, 11	8, 11	Matching reference profile locus
D16S539	9	9	Matching reference profile locus
D2S1338	17, 19	17, 19	Matching reference profile locus
D19S433	13, 14	13, 14	Matching reference profile locus
vWA	16, 18	16, 18	Matching reference profile locus
TPOX	8, 10	8, 10	Matching reference profile locus
D18S51	17, 18	17, 18	Matching reference profile locus
Amelogenin*	X	X	Not interpreted
D5S818	12, 13	12, 13	Matching reference profile locus
FGA	22, 23	22, 23	Matching reference profile locus

*Amelogenin was excluded from interpretation, as analyses focused on autosomal STR loci.
