# Supplementary material for: STR analysis of human DNA recovered from bathwater and other water samples for forensic identification
Source: PLoS One. 2026 Mar 25;21(3):e0345878. doi: 10.1371/journal.pone.0345878 (PMC13016345; doi:10.1371/journal.pone.0345878)
Supplement: S6 Table — (PDF) [file pone.0345878.s006.pdf]

S6 Table. Locus-by-locus STR interpretation for a representative bathwater sample collected after 5 minutes of immersion (Volunteer no. 5).

Locus	Reference genotype	Observed in bathwater	Interpretation
D8S1179	12, 15	12, **13**, 15	**Allelic mixture**
D21S11	30, 31	**29**, 30, 31	**Allelic mixture**
D7S820	11, 12	11, 12	Matching reference profile locus
CSF1PO	10, 13	10, 13	Matching reference profile locus
D3S1358	15, 16	15, 16	Matching reference profile locus
TH01	7, 8	**6**, 7, 8	**Allelic mixture**
D13S317	8, 11	8, 11	Matching reference profile locus
D16S539	9, 10	9, 10	Matching reference profile locus
D2S1338	20, 26	**19**, 20, 26	**Allelic mixture**
D19S433	13	13	Matching reference profile locus
vWA	14, 16	14, 16	Matching reference profile locus
TPOX	9, 11	9, 11	Matching reference profile locus
D18S51	14, 15	**14**, 15, 19	**Allelic mixture**
Amelogenin*	X	X, **Y**	Not interpreted
D5S818	9, 11	9, 11	Matching reference profile locus
FGA	18, 24	18, 24	Matching reference profile locus

*Amelogenin was excluded from interpretation, as analyses focused on autosomal STR loci.
Allelic mixtures (bold characters) were defined as loci containing a mixture of the bather's alleles and those from other individuals.
Formatting (bold and italic characters) was used in tables to improve readability; corresponding features are indicated by arrows and labels in electropherograms.
