# Supplementary material for: STR analysis of human DNA recovered from bathwater and other water samples for forensic identification
Source: PLoS One. 2026 Mar 25;21(3):e0345878. doi: 10.1371/journal.pone.0345878 (PMC13016345; doi:10.1371/journal.pone.0345878)
Supplement: S7 Table — (PDF) [file pone.0345878.s007.pdf]

S7 Table. Locus-by-locus STR interpretation for a representative bathwater sample collected after 1 minute of immersion (Volunteer no. 5).

Locus	Reference genotype	Observed in bathwater	Interpretation
D8S1179	12, 15	12, (14), 15	(Allele drop-in)
D21S11	30, 31	**29**, 30, 31	**Allelic mixture**
D7S820	11, 12	11, 12	Matching reference profile locus
CSF1PO	10, 13	10, 13	Matching reference profile locus
D3S1358	15, 16	15, 16	Matching reference profile locus
TH01	7, 8	7, 8	Matching reference profile locus
D13S317	8, 11	8, 11	Matching reference profile locus
D16S539	9, 10	9, 10	Matching reference profile locus
D2S1338	20, 26	20, 26	Matching reference profile locus
D19S433	13	13	Matching reference profile locus
vWA	14, 16	14, 16	Matching reference profile locus
TPOX	9, 11	9, 11	Matching reference profile locus
D18S51	14, 15	15, 19	Matching reference profile locus
Amelogenin*	X	X	Not interpreted
D5S818	9, 11	9, 11	Matching reference profile locus
FGA	18, 24	18, 24	Matching reference profile locus

*Amelogenin was excluded from interpretation, as analyses focused on autosomal STR loci.
Allele drop-ins (in parentheses) were defined as alleles not attributable to either the bather or any known individual.
Allelic mixtures (bold characters) were defined as loci containing a mixture of the bather's alleles and those from other individuals.
