# Supplementary material for: STR analysis of human DNA recovered from bathwater and other water samples for forensic identification
Source: PLoS One. 2026 Mar 25;21(3):e0345878. doi: 10.1371/journal.pone.0345878 (PMC13016345; doi:10.1371/journal.pone.0345878)
Supplement: S8 Table — (PDF) [file pone.0345878.s008.pdf]

S8 Table. Locus-by-locus STR interpretation for a representative bathwater sample collected prior to immersion (Volunteer no. 8).

Locus	Reference genotype	Observed in bathwater	Interpretation
D8S1179	10	10	Matching reference profile locus
D21S11	28, 32.2	Not detected	Allelic non-detection
D7S820	12, 13	Not detected	Allelic non-detection
CSF1PO	11, 12	Not detected	Allelic non-detection
D3S1358	15	*16*	*Only non-bather locus*
TH01	7, 9	Not detected	Allelic non-detection
D13S317	9	Not detected	Allelic non-detection
D16S539	10, 12	Not detected	Allelic non-detection
D2S1338	16, 23	Not detected	Allelic non-detection
D19S433	14	Not detected	Allelic non-detection
vWA	16, 17	Not detected	Allelic non-detection
TPOX	8, 11	Not detected	Allelic non-detection
D18S51	16, 19	Not detected	Allelic non-detection
Amelogenin*	X	*Y*	Not interpreted
D5S818	12, 13	Not detected	Allelic non-detection
FGA	22, 23	*20*	*Only non-bather locus*

*Amelogenin was excluded from interpretation, as analyses focused on autosomal STR loci.
Only non-bather loci (*italic characters*) were defined as loci containing only alleles not attributable to the bather, based on comparison with the bather's reference profile.
RFU values are shown only for detected alleles, loci with no detectable peaks are indicated as "Not detected"' and interpreted as "Allelic non-detection"..
