# Supplementary material for: STR analysis of human DNA recovered from bathwater and other water samples for forensic identification
Source: PLoS One. 2026 Mar 25;21(3):e0345878. doi: 10.1371/journal.pone.0345878 (PMC13016345; doi:10.1371/journal.pone.0345878)
Supplement: S9 Table — Note: Two independent runs were performed for each volunteer or water sample, with duplicate measurements per run. Values marked as N.d. (not detected) were excluded from all calculations. Representative electropherograms (S2–S5 Figs) and their corresponding locus-by-locus interpretations are provided in S5–S8 Tables. Quantitative evaluation of minor allelic peaks interpreted as allelic mixtures or allele drop-ins, including peak heights (RFU), stutter ratios, and comparison with locus-specific −1 stutter filter percentages, is provided in S9 Table. (PDF) [file pone.0345878.s009.pdf]

S9 Table. Peak height (RFU) values and stutter ratio assessment for alleles interpreted as drop-ins or allelic mixtures shown in S3 and S4 Figures.

Figure No.	Locus	Minor allele	Minor allele peak height (RFU)	Adjacent true allele peak height (RFU)	Position relative to true allele	Stutter ratio (%)	Kit stutter filter (%)	Interpretation
S3 Figure	D8S1179	13	226	8264	+1	2.73	N/A	Allelic mixture (+1 position; intepreted cautiously)
	D21S11	29	561	3719	-1	15.08	10.67	Allelic mixture
	TH01	6	346	5797	-1	5.97	4.08	Allelic mixture
	D2S1338	19	366	2814	-1	13.01	12.44	Allelic mixture
	D18S51	19	175	-	-	N/A	N/A	Allelic mixture (Non-stutter position)
S4 Figure	D8S1179	14	345	2755	-1	12.52	10.32	Allele drop-in
	D21S11	29	257	2120	-1	12.12	10.67	Allelic mixture

A stutter peak refers to a minor peak one repeat smaller (-1) or one repeat larger (+1) than the adjacent true allele.
Stutter ratios were calculated as (minor peak height / true allele peak height) x 100.
Kit stutter filter (%) refers to locus-specific -1 stutter filter percentages for the AmpFlSTR Identifiler Plus PCR Amplification Kit.
N/A indicates not applicable (e.g., non-stutter positions where no stutter comparison was possible).
Minor peaks detected above the analytical threshold (175 RFU) with no corresponding adjacent true allele peak were evaluated separately.
Locus-specific stutter filter percentages were obtained from the AmpFlSTR Identifiler Plus PCR Amplification Kit User Guide (Thermo Fisher Scientific), Table 5.
