# Supplementary figures and images for: STR analysis of human DNA recovered from bathwater and other water samples for forensic identification
Source: PLoS One. 2026 Mar 25;21(3):e0345878. doi: 10.1371/journal.pone.0345878 (PMC13016345; doi:10.1371/journal.pone.0345878)

**DD**

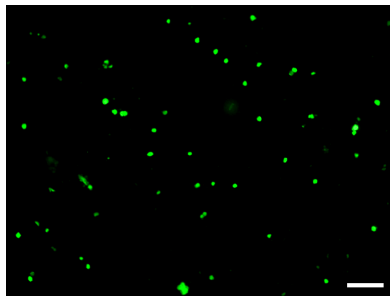

**H&E**

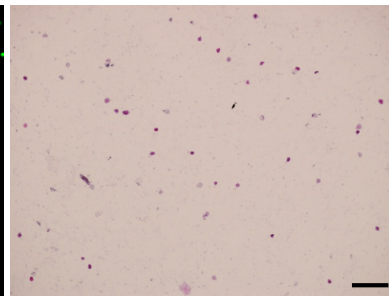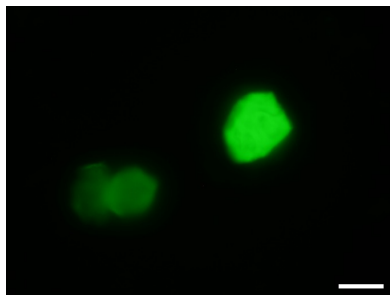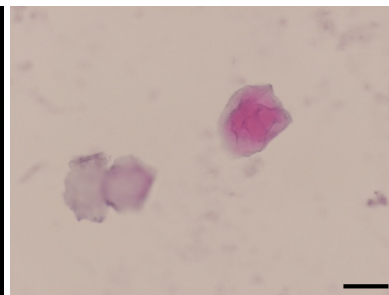

Supplement: S1 Fig — Upper panels show low-magnification views, and lower panels show high-magnification views. Scale bars: 200 µm (upper), 20 µm (lower). Shed cells were recovered during the final stage of bathwater filtration (at 3–4 mL remaining from each 250 mL fraction) and concentrated to 500 µL using a vacuum dryer. (PDF) [file pone.0345878.s010.pdf]

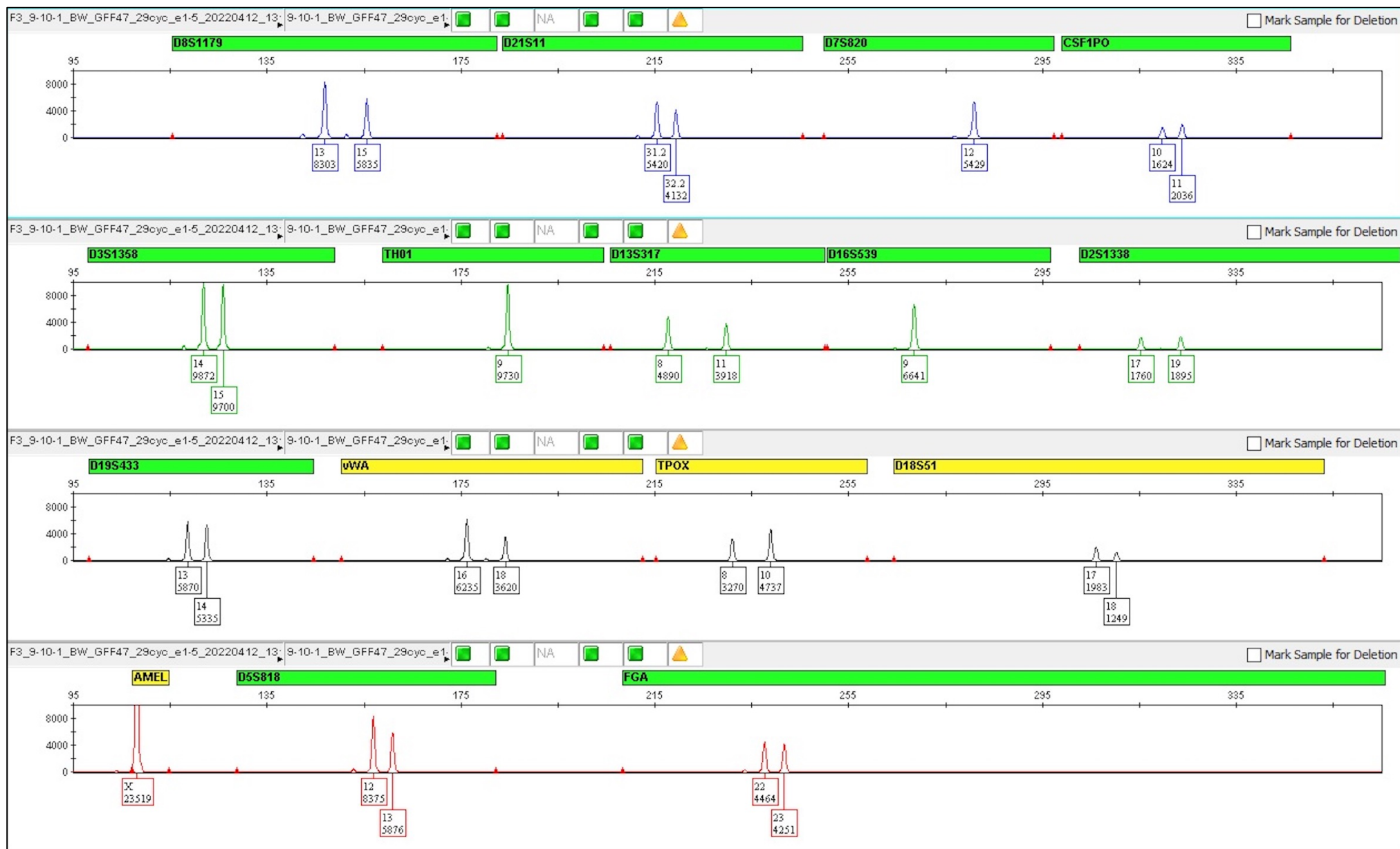

Supplement: S2 Fig — All autosomal STR loci matched the reference profile of the bather, indicating a complete STR profile. Amelogenin was excluded from interpretation, as analyses focused on autosomal STR loci. (PDF) [file pone.0345878.s011.pdf]

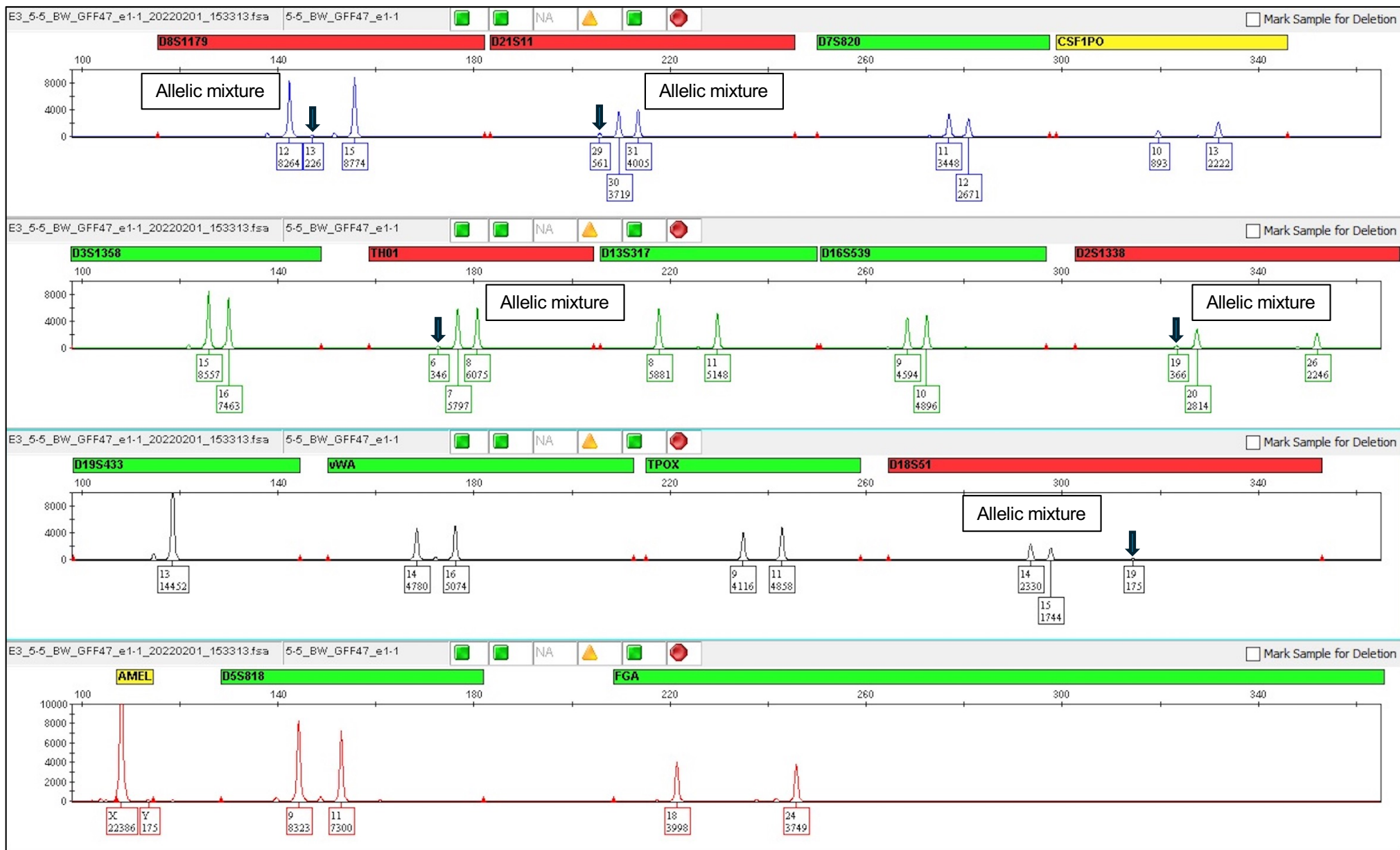

Supplement: S3 Fig — Arrows indicate alleles not attributable to the bather, consistent with the definition of allelic mixtures. (PDF) [file pone.0345878.s012.pdf]

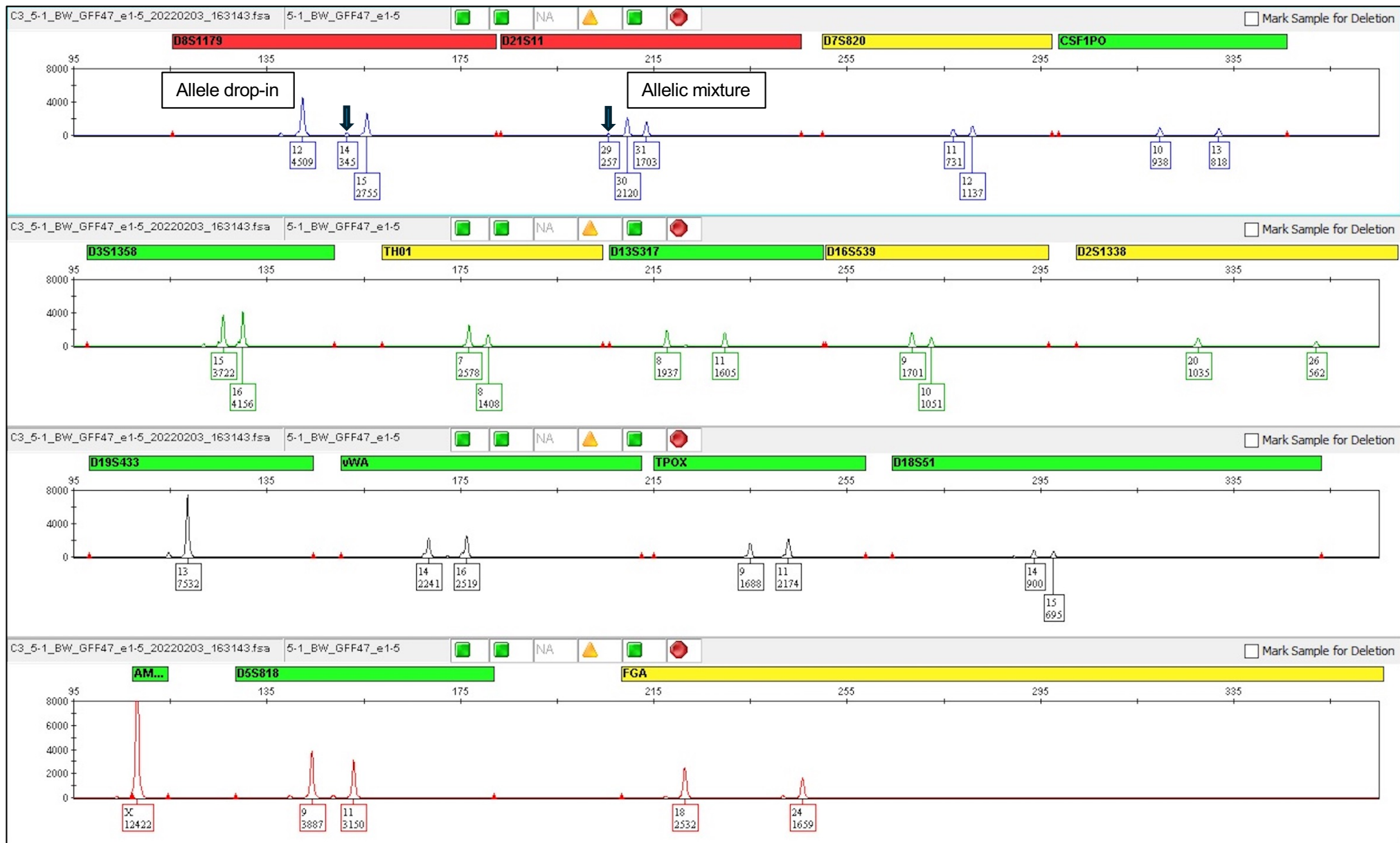

Supplement: S4 Fig — Arrows indicate alleles not attributable to the bather (Allelic mixtures) and alleles not attributable to either the bather or any known individual (Allele drop-ins). (PDF) [file pone.0345878.s013.pdf]

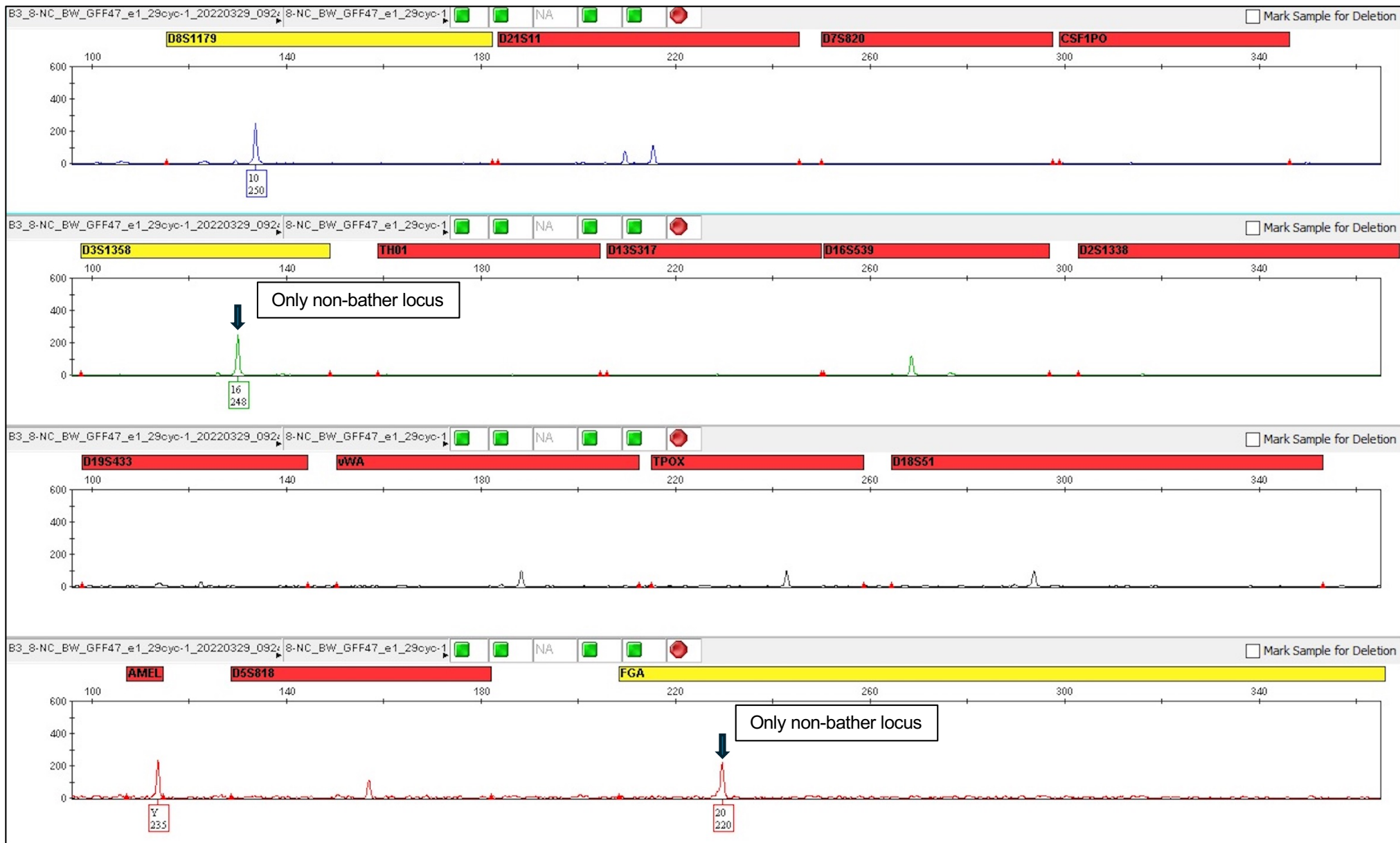

Supplement: S5 Fig — Arrows indicate alleles not attributable to the bather. Loci at which only non-bather alleles were detected are indicated below the corresponding loci. (PDF) [file pone.0345878.s014.pdf]
